# Afghan migrants face more suboptimal care than natives: a maternal near-miss audit study at university hospitals in Tehran, Iran

**DOI:** 10.1186/s12884-017-1239-2

**Published:** 2017-02-13

**Authors:** Soheila Mohammadi, Soraya Saleh Gargari, Masoumeh Fallahian, Carina Källestål, Shirin Ziaei, Birgitta Essén

**Affiliations:** 1 Department of Women’s and Children’s Health, International Maternal and Child Health (IMCH), Akademiska sjukhuset, Uppsala University , Uppsala, SE-751 85 Sweden; 2 Infertility and Reproductive Health Research Center, Shahid Beheshti University of Medical Sciences, Tehran, Iran

**Keywords:** Maternal near miss, Audit, Quality of care, Afghan migrants, Preventability, Iran

## Abstract

**Background:**

Women from low-income settings have higher risk of maternal near miss (MNM) and suboptimal care than natives in high-income countries. Iran is the second largest host country for Afghan refugees in the world. Our aim was to investigate whether care quality for MNM differed between Iranians and Afghans and identify potential preventable attributes of MNM.

**Methods:**

An MNM audit study was conducted from 2012 to 2014 at three university hospitals in Tehran. Auditors evaluated the quality of care by reviewing the hospital records of 76 MNM cases (54 Iranians, 22 Afghans) and considering additional input from interviews with patients and professionals. Main outcomes were frequency of suboptimal care and the preventable attributes of MNM. Crude and adjusted odds ratios with confidence intervals for the independent predictors were examined.

**Results:**

Afghan MNM faced suboptimal care more frequently than Iranians after adjusting for educational level, family income, and insurance status. Above two-thirds (71%, 54/76) of MNM cases were potentially avoidable. Preventable factors were mostly provider-related (85%, 46/54), but patient- (31%, 17/54) and health system-related factors (26%, 14/54) were also important. Delayed recognition, misdiagnosis, inappropriate care plan, delays in care-seeking, and costly care services were the main potentially preventable attributes of MNM.

**Conclusions:**

Afghan mothers faced inequality in obstetric care. Suboptimal care was provided in a majority of preventable near-miss events. Improving obstetric practice and targeting migrants’ specific needs during pregnancy may avert near-miss outcomes.

## Background

Disparities in maternal outcomes between migrants from low-income settings and natives in high-income countries are well documented in the literature [1, 2]. Migrants’ vulnerability to poor pregnancy outcomes is partly explained by individual high-risk profiles such as comorbidities and socioeconomic disadvantages [3, 4]. Furthermore, suboptimal care due to incongruent language and communication barriers, and unequal access to obstetric services are more frequent in migrant populations than European natives [3, 5].

Iran, with 79 million inhabitants, hosts refugees from neighbouring countries and accommodates an estimated one million registered and over two million unregistered migrants from Afghanistan [6, 7]. Essential interventions have been scaled up in this country and institutional delivery of 95% and a 75% reduction in maternal mortality ratio (25 per 100,000 live births) between 1990 and 2015 indicate healthcare achievements [8, 9]. Iran has faced international sanctions and financial constraints in recent decades and health resources have been inadequate to provide universal coverage for public health [10]. Therefore, in-patient care was covered by health insurance for 90% of Iranians, while migrants were uninsured before the initiation of the new health system reform and public access to the Salamat Insurance Scheme in 2015 [7, 10]. Our recent study in Tehran showed an increased risk of maternal near miss (MNM) among Afghan migrants through a lack of health insurance [11]. Iranians and Afghans have similar religious backgrounds and the majority of Afghans speak a language (Dari) similar to that of their hosts (Farsi). Therefore, the communication barriers to optimal care for migrants described in high-income settings appear to be less applicable in this context [3, 12].

MNM is defined as a woman who survives life-threatening conditions during pregnancy, childbirth, or within six weeks postpartum [13]. As high-quality care is crucial in saving lives, World Health Organization (WHO) developed a MNM audit approach in 2011 to routinely evaluate obstetric care quality in health facilities [13].

This study aimed at evaluating whether care quality differed between Iranian and Afghan mothers with near-miss morbidity using the WHO approach. Additionally, we sought to identify potentially preventable factors predisposing to MNM.

## Methods

This audit study was part of an MNM project that was conducted between March 2012 and May 2014 at three hospitals affiliated with the Shahid Beheshti University of Medical Sciences in Tehran. Approximately 140 public and private hospitals provide care services in Tehran and the caesarean section (CS) rate stands at 74% [14]. Antenatal visits are included in primary health care and are provided free of charge for both natives and migrants.

Study sites were a secondary hospital with over 4,500 annual births and two tertiary referral hospitals with over 600 and 1,000 deliveries per year, respectively. The obstetric wards were staffed with 21 consultants and 68 residents in obstetrics and gynaecology, and 46 midwives. Consultants, residents, and midwives provide 24-hour medical staffing using national and local guidelines for obstetric care provision. Residents, under supervision of consultants, are responsible for all deliveries regardless of risk and nationality. The study hospitals provided intensive care services for adults and neonates.

**Fig. 1:**
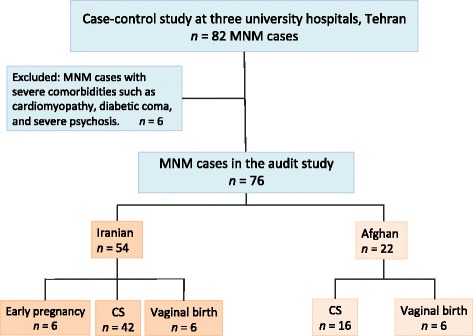
The flow diagram of the study population for maternal near-miss audit at three university hospitals in Tehran, Iran, 2012 to 2014

At each hospital, one consultant and one resident were selected as research group members. The residents identified MNM cases under supervision of the consultants during daily morning reports. The WHO MNM criteria were used to identify cases prospectively during the first phase of the MNM project [13]. However, these criteria were modified slightly in the case of two indicators in order to minimise inappropriate exclusion of women whose lives were seriously endangered due to obstetric complications but who did not fulfil the WHO criteria due to a limited institutional resources [13]. As the secondary hospital had limited access to blood products (Rh-negative blood types in particular), modified criteria were the administration of four or more units of blood products and a rapid reduction of platelet count to below 75,000 platelets/ml. An acute decrease of ≥4 g/dl in the haemoglobin concentration was also applied as a near-miss criterion. Figure 1 shows the study population.

Maternal age (<20, 20–34, ≥35 years), parity (0, 1–2, ≥3 para), and body mass index (BMI) (<18.5: underweight, 18.5–24.9: normal weight, ≥25: overweight and obese) were considered as maternal factors. Level of education (illiteracy and primary, secondary and higher), family income (low if it barely covered household expenditures, medium if it paid such bills, and high if it was more than self-reported expenses), health insurance (representing occupational status of the woman or her husband), and nationality (Iranian and Afghan according to country of birth) were identified as socioeconomic factors. Antenatal care coverage (at least four visits), timing of the near-miss event (upon or after arrival), admission status (primary or referral), previous CS, CS delivery in present pregnancy, night-shift delivery (2 o’clock pm till 8 o’clock am), severe anaemia (haemoglobin ≤10 g/dl), and comorbidity (diabetes, chronic hypertension, haematological disorders, previous pelvic operation) were assessed as medical factors.

### Audit procedure

The main researcher primarily reviewed patients’ records, including admission, operation, and nurses’ notes, ordering sheets, laboratory and pathology reports, and summary notes. For each case, a research form was completed with all background data including obstetric history, and clinical data related to near-miss events, as well as a copy of cardiotocoghraphy (CTG) traces and other available documents.

**Fig. 2:**
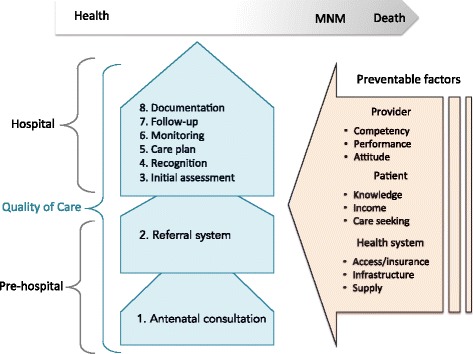
Audit framework for evaluating the quality of obstetric care and preventability for 76 maternal near misses at three university hospitals in Tehran, Iran, 2012 to 2014

Three auditors, a Maternal Foetal Medicine physician and two board-certified obstetricians, comprised the audit team. During the study period, the main researcher organised audit panels and presented case histories anonymously (in terms of identity, nationality, and the hospital) to these panels. Auditors used a conceptual framework (Fig. 2) and performed individual case note review to evaluate obstetric care quality and preventability of near-miss events. Additionally, when documentation was insufficient for making clinical judgments, the main researcher interviewed those professionals identified as being the care providers responsible for the particular case to obtain clinical information. Thirdly, 24 near-miss mothers (16 in-person and 8 phone calls) were interviewed to provide additional ideas and input. For each case, the quality of eight care items was audited and the final decision was achieved by consensus among the three auditors (case exemplars are shown in Tables 1, 2 and 3). Antenatal care and referral systems (two items) were assessed to evaluate the quality of pre-hospital care. The quality of hospital care was analysed using a systematic approach based on six items: initial assessment, recognition, appropriate care plan, monitoring of critical conditions, provision of adequate information to women at discharge and planned follow-up, as well as proper documentation [15]. Pre-hospital obstetric care was labelled suboptimal if either antenatal care or referral system was inadequate (≥50% of items). Hospital care was considered suboptimal when three or more of those six items (≥50%) were inadequate. It was possible for one woman with near-miss morbidity to experience several inadequate items and preventable factors during the period of her hospitalisation.

**Table 1:** Maternal, socioeconomic, and medical background factors among 54 Iranian and 22 Afghan near misses at three university hospitals in Tehran, Iran, 2012 to 2014

Background factors	Iranian 54 (%)	Afghan 22 (%)	*p-*value
Maternal			
Age (years)			
<20	2 (4)	3 (14)	
20–34	36 (67)	17 (77)	
≥35	16 (29)	2 (9)	0.06
Parity			
0	21 (39)	7 (32)	
1–2	27 (50)	9 (41)	
≥3	6 (11)	6 (27)	0.10
Body mass index (kg/m2)			
Underweight <18.5	4 (7)	1 (5)	
Normal weight 18.5–24.9	23 (43)	15 (68)	
Over weight and obese ≥25	27 (50)	6 (27)	0.08
Socioeconomic			
Education			
Illiteracy and primary	23 (43)	17 (77)	0.01^a^
Secondary and higher	31 (57)	5 (23)	
Family income			
Low	23 (43)	20 (91)	0.01^a^
Medium and high	31 (57)	2 (9)	
Insurance	43 (80)	0	0.01^a^
Medical			
Antenatal care coverage^b^	49 (96)	18 (82)	0.07
Near miss upon arrival	36 (67)	11 (50)	0.20
Primary admission	38 (70)	11 (50)	0.10
Previous caesarean delivery	19 (35)	8 (36)	0.90
Caesarean delivery^c^	42 (87)	16 (73)	0.10
Night-shift delivery	33 (69)	17 (77)	0.50
Severe anaemia^d^	10 (18)	5 (23)	0.70
Comorbidity^e^	25 (46)	11 (50)	0.80

^a^Significant chi-square test

^b^Not applicable for three MNM cases with ectopic pregnancy (49/51)

^c^Not applicable for six MNM cases in early pregnancy

^d^Haemoglobin ≤10 g/dl

^e^Includes diabetes, haematological disorders, epilepsy, chronic hypertension, previous pelvic operation

**Table 2:** Severe complications and near-miss events in 54 Iranian and 22 Afghan near-miss cases at three university hospitals in Tehran, Iran, 2012 to 2014

Conditions	Iranian *n* (%)	Afghan *n* (%)	Total *n* (%)	*p-*value
Severe obstetric complications				
Severe postpartum haemorrhage	17 (31)	11 (50)	28 (37)	0.40
Severe pre-eclampsia and eclampsia	17 (31)	9 (41)	26 (34)	0.50
Placenta previa/abnormal invasive placenta	7 (13)	1 (4)	8 (10)	0.30
Placental abruption	3 (5)	2 (9)	5 (7)	0.60
Ectopic pregnancy	5 (9)	0	5 (7)	n/a
Abortion	1 (2)	0	1 (1)	n/a
Obstetric haemorrhage^a^	2 (4)	2 (9)	4 (5)	0.20
Puerperal sepsis	4 (7)	2 (9)	6 (8)	0.80
Pulmonary embolism	2 (4)	0	2 (3)	n/a
Other^b^	6 (11)	4 (18)	10 (13)	0.40
Organ dysfunctions (near-miss events)				
Coagulation and haematological	53 (98)	22 (100)	75 (99)	0.10
Cardiovascular	10 (22)	8 (35)	18 (26)	0.80
Uterine/hysterectomy	9 (18)	5 (23)	14 (18)	0.30
Respiratory	10 (18)	2 (9)	12 (16)	0.20
Renal	4 (7)	2 (9)	6 (9)	0.80
Neurological	3 (5)	1 (5)	4 (6)	0.90
Hepatic	0	1 (5)	1 (1)	n/a
Admission to intensive care unit	36 (67)	14 (64)	50 (66)	0.80
Laparotomy/reoperation	3 (5)	5 (23)	8 (10)	0.04

^a^Excessive bleeding because of an abnormality in the process of childbirth

^b^Includes epilepsy, acute fatty liver, haematological disorders

**Table 3:** Statistical models of the associations between suboptimal care and socioeconomic factors of 76 maternal near misses at three university hospitals in Tehran, Iran, 2012 to 2014

Socioeconomic factors	OR^a^ (95% CI) Model 1^b^	AOR (95% CI) Model 2^c^	AOR (95% CI) Model 3^d^
Education			
Illiteracy and primary	2.9 (1.1–7.6)	2.0 (0.5–7.8)	1.6 (0.4–6.2)
Secondary and higher	1.00	1.00	1.00
Family income			
Low	3.4 (1.3–9.0)	1.1 (0.2–4.9)	1.4 (0.3–5.7)
Medium and high	1.00	1.00	1.00
Nationality			–
Afghan	6.3 (1.7–23.9)	4.4 (1.1–18.2)	
Iranian	1.00	1.00	
Insurance	0.2 (0.1–0.6)	–	–
Insurance-nationality		–	
Uninsured Afghan	7.3 (1.9–28.3)		5.1 (1.2–22.6)
Uninsured Iranian	2.0 (0.5–7.9)		1.7 (0.4–7.1)
Insured Iranian	1.00		1.00

^a^Odds ratio

^b^Crude OR for socioeconomic factors

^c^Adjusted OR for education, family income, and nationality

^d^Adjusted OR for socioeconomic factors while “insurance-nationality” substitutes for insurance and nationality

In addition, auditors discussed preventability of near-miss events and agreed on the potential factors that could have prevented MNM or minimised the severity of the events at three levels: provider, patient, and health system [16, 17].

### Statistical analysis

Chi-square test was used to examine and compare background factors, severe complications, and near-miss events between Iranians and Afghans. Statistical Package for the Social Sciences software, Version 21, was used for statistical analysis. Differences were considered significant where a probability of less than 0.05 was found. The association between suboptimal care and those factors that were significantly different between Iranians and Afghans was assessed by crude and adjusted odds ratios (OR, AOR) in three models. Health insurance was found protective against MNM and all Afghans and a number of Iranians were uninsured [11]. Therefore, a new independent variable was created and named “insurance-nationality” with three categories: insured Iranian, uninsured Iranian, and uninsured Afghan to examine the effect of lacking insurance on receiving suboptimal care for both nationalities. Model 1 represented crude associations between suboptimal care and maternal education, family income, nationality, insurance, and insurance-nationality. In Model 2, maternal education, family income, and nationality were taken into account to identify independent predictors of suboptimal care. In Model 3, “insurance-nationality” was added as a substitute for “nationality” in Model 2. The independent variables were controlled for collinearity.

## Results

Table 1 and 2 show a comparison between women’s background factors and severe obstetric complications as well as near-miss events for Iranians and Afghans. Socioeconomic factors in women’s background variables of 76 cases, differed significantly between Iranians and Afghans. Severe postpartum haemorrhage and severe preeclampsia/eclampsia were the most frequent obstetric complications that both MNM groups experienced. On average, each Iranian and Afghan woman developed 2.3 and 2.6 near-miss events, respectively.

### Audit findings

Generally, inadequate care items were identified in 85% (65/76) of cases. The majority of cases (62%, 47/76) had already developed a critical condition upon arrival or developed a near-miss event within six hours of admission. The proportion of inadequate items for pre-hospital care was similar for both nationalities. However, inadequate items for hospital care were more frequent among Afghan cases than Iranians [OR 2.7, 95% confidence interval (CI): 1.8–4.1].

Suboptimal care was identified in 70% (38/54) of Iranians and 86% (19/22) of Afghans. While suboptimal care at pre-hospital level was similar for both nationalities (OR 2.2, 95% CI: 0.8–6.0), at the hospital it was disproportionately more common in Afghan cases (OR 4.5, CI: 1.3–15.0). Model 1 in Table 3 shows that being illiterate or having primary education, low-income status, and being Afghan increased odds of receiving suboptimal care. On the contrary, health insurance protected mothers against suboptimal care. The association between suboptimal hospital care and Afghan nationality remained significant after adjusting for maternal education and income (Model 2 in Table 3) and even after additional adjustment for the effect of health insurance (Model 3 in Table 3).

Auditors determined that 71% (54/76) of MNM cases had at least one near-miss event that could have potentially been prevented. Preventability of near-miss events was not different between Iranians and Afghans (67%, 36/54 versus 81%, 18/22; *p-*value: 0.2). As Table 4 shows, although provider-related factors were involved in majority of cases with preventable events, patient— and health system-related factors could also prevent the development of MNM. The following sections describe related examples of missed opportunities to optimal care.

**Table 4:** Potentially preventable factors at three levels that attributes to 54 maternal near misses (36 Iranians and 18 Afghans) at university hospitals in Tehran, Iran, 2012 to 2014

Preventable factors	Iranian *n* (%)	Afghan *n* (%)	Total *n* (%)
Provider level	30 (83)	16 (89)	46 (85)
Inadequate antenatal care	7 (19)	3 (17)	10 (18)
Delayed referring/before stabilization	5 (14)	3 (17)	8 (15)
Inadequate initial assessment	15 (42)	10 (55)	25 (46)
Delayed recognition/misdiagnosis	15 (42)	16 (89)	31 (57)
Inappropriate care plan	23 (64)	11 (61)	34 (63)
Inadequate postpartum monitoring	4 (11)	3 (17)	7 (13)
Patient level	10 (28)	7 (39)	17 (31)
Inadequate knowledge	2 (5)	2 (11)	4 (7)
Financial constraints	3 (8)	3 (17)	6 (11)
Delayed care-seeking	5 (14)	3 (17)	8 (15)
Non-compliance with recommendation	4 (11)	4 (22)	8 (15)
Health system level	8 (22)	6 (33)	14 (26)
Costly care services	3 (8)	4 (22)	7 (13)
Non-functional referrals	1 (3)	1 (5)	2 (4)
Medication shortage	3 (8)	1 (5)	4 (7)
Unavailable intensive care unit beds	1 (3)	2 (11)	3 (5)

In 82% (23/28) of MNM cases of severe postpartum haemorrhage, the amount of blood loss was neither estimated nor adequately assessed. Moreover, delayed recognition of the severity of haemorrhage and inadequate stepwise management were identified in 57% (16/28) of cases. Initial assessment in 61% (16/26) of MNM cases of hypertensive disorders was inadequate. Emergency CS due to severe pre-eclampsia was performed in 21 cases, of which 71% (15/21) occurred before stabilisation and treatment of severe hypertension. While placenta previa was the third most common obstetric complication (10%, 8/76) that led to near-miss morbidity, pre-surgical evaluation and the decision process for adopting a surgical approach was missed in 50% of cases.

Three women with preterm pregnancy and co-existing epilepsy arrived at hospital with uncontrollable fits due to either discontinuation of treatment or irregular use of anticonvulsive therapy. They were delivered by emergency CS on suspicion of eclampsia while adequate history taking and initial assessment did not take place. Audits of CTG traces could only confirm abnormal CTG in 20% of those near-miss cases that delivered by CS due to foetal distress. There was no CTG documentation confirming foetal distress in 60% of cases and the opportunity for adequate assessment of CTG trace was missed in the remaining 20%. Tables 5, 6 and 7 present examples of MNM cases and the related clinical judgements.

**Table 5:** Example of missed opportunities linked to care items for obstetric haemorrhage

Case 1	A 21-year-old Afghan mother, 0P, in 38 weeks of gestation, was admitted to hospital with labour pains in latent phase. She was delivered by emergency CS due to foetal distress on the day shift. Ten hours after operation she was pale, had pre-shock status, and the reported haemoglobin level was 7.4 g/dl. Re-operation was performed, a very large hematoma in left broad ligament was detected, and 12 units of different blood products were transfused. She went back to the hospital two weeks after discharge due to fever and haematuria. Further examination revealed left ureter injury.
Care items	Audit findings
Initial assessment	Foetal heart rates were monitored and assessed inadequately.
Recognition	No evidence was found to agree foetal distress. Intra-abdominal hematoma was recognised with delay.
Care plan	The indicated evidence for emergency CS was missing.
Monitoring	Postpartum controls early after CS were not documented and were inadequate for early detection of intra-abdominal bleeding.
Preventability	Near-miss events (decreased haemoglobin, re-operation, blood transfusion) and the injured ureter could have potentially been prevented by better obstetric practice (provider-related).

**Table 6:** Example of missed opportunities linked to care items for placenta previa

Case 2	A 36-year-old native mother, 2P, with two previous CS was admitted to hospital due to low back pain in 39 + 3 weeks of gestation. According to ultrasound examinations during antenatal visits, she had low-lying placenta previa. Emergency CS was performed two hours after admission on the night shift and the operation ended up with CS hysterectomy due to abnormal invasive placenta. More than 20 units of blood products were transfused, the mother was admitted at intensive care unit and had long-lasting intubation. Pathologic examination of uterus specimen revealed placenta increta.
Care items	Audit findings
Antenatal care	Despite two previous CS and low-lying placenta previa, examination of placental orientation for better obstetric plan during pregnancy was not conducted. Despite repeat CS and previa, no elective surgery was planned.
Referral system	No timely referral from antenatal clinic to the hospital was made.
Initial assessment	Despite risk for abnormal invasive placenta, no assessment at hospital was performed.
Recognition	Recognition of abnormally invasive placenta in a high-risk mother was missed before operation room.
Care plan	No evidence was found indicating acute CS on the night shift for a high-risk surgery.
Documentation	Estimation of blood loss during operation was not documented. Near-miss events such as the amount of administered blood, admission to intensive care unit, and long-lasting intubation were not documented in summary notes.
Preventability	The near-miss events could have potentially become less critical and traumatic for woman and her family by better obstetric practice (provider-related).

**Table 7:** Example of missed opportunities linked with sepsis and postpartum haemorrhage

Case 3	A 24-year-old Afghan mother, 3P, was admitted to hospital one week after home delivery with long-lasting bleeding, weakness, and high fever. She was in pre-shock status and the reported haemoglobin level was 5.6 g/dl. She was resuscitated with blood transfusion and was treated with intravenous antibiotic due to postpartum endometritis. Ultrasound examination was done after three days and retained placenta was detected. Fever went down after evacuation and curettage and she chose to leave the hospital before doctor’s recommendation. She was interviewed afterward and said that the family could not afford the cost of hospital obstetric services.
Care items	Audit findings
Initial assessment	Despite home delivery and the risk of retained placenta, history taking and initial examination of uterus cavity were incomplete.
Recognition	Delayed recognition of retained placenta in a mother with anaemia and postpartum endometritis was identified.
Care plan	The management of postpartum endometritis and retained placenta were inappropriate.
Documentation	Previous obstetric history and risk of postpartum haemorrhage, antenatal visits and delivery process at home for index pregnancy were not documented.
Preventability	The near-miss events could have potentially prevented by affordable safe childbirth (health system) and timely care-seeking (patient). Hospital care was also suboptimal.

## Discussion

Our findings demonstrate that obstetric care at hospital was more suboptimal for Afghan MNMs than Iranians. Moreover, care providers by optimal performance could potentially prevent a majority of near-miss events.

In contrary to literature, differences in background profiles, socioeconomic factors, language, and religion between Iranians and Afghans could hardly explain our findings [4, 16, 18]. Having a low level of education is a well-known association with increased risk of adverse maternal outcomes by delayed care-seeking and poor compliance with treatments [19]. All Afghans in the present study were uninsured and had a lower level of education compared to Iranians; however, the proportion of near misses upon arrival did not differ between Iranians and Afghans. While suboptimal care was more probable for uninsured Afghans, this probability was not found for uninsured Iranians. Moreover, having insurance coverage in European countries is inadequate to protect immigrants from disproportionate suboptimal care [5, 12]. Social differentiation, isolation, and women’s self-perceptions can dissociate mothers from adequate care [20]. These factors among Afghans could affect obstetric care quality but this study was unable to elaborate on such factors.

In accordance with prior studies, we found that care providers had fundamental roles in the prevention of near-miss morbidity by making timely diagnosis and successful management of obstetric complications [16, 21]. Auditors noticed that although guidelines were accessible to care professionals, medical management poorly adhered to them. Literature suggests that in addition to establishing guidelines, optimal care provision requires providers’ beliefs and institutional support [22]. Conducting audits in obstetrics can provide practical measures for tackling care deficiencies and may stimulate tailored interventions to improve maternal care quality [23, 24].

In agreement with other publications, we found that patient- and health system-related factors were involved in preventable near-miss events [3, 16]. For instance, home delivery or delayed care-seeking that may result from financial constraints at patient level could have been avoided by insurance coverage at the level of health system. Although healthy motherhood is part of women’s rights to health and life, poverty seriously affects these rights when the needed care is available but unaffordable [25].

To our knowledge, the present study is the first attempt to apply the WHO MNM audit in Iran to evaluate care quality within a migration perspective. Using the WHO criteria was feasible to identify MNM cases prospectively in our setting, while the MNM tool failed to identify all eligible cases within a national data in the Netherlands, retrospectively [26]. Collecting first-hand data from near-miss survivors and care providers was a real strength in our study and disclosed valuable inputs that might not have been identified by reviewing the case notes alone. The audit framework enabled us to identify major obstacles to optimal care provision and facilitated determining barriers against accessing to such care. Moreover, the classification of potential preventable attributes of MNM into provider-, patient-, and health system-related factors might have a qualitative value in revealing where and how the tailored interventions could avert near-miss outcomes [17]. As missed opportunities to better manage severe complications were found repeatedly at all of the study hospitals, the identified quality might mirror those of other university hospitals in Tehran.

The main limitation of this audit study is that our findings are based on a small sample of MNM cases. As the WHO near-miss approach underlines, the optimum number of cases for evaluating care quality has not been established [13]. Our small sample could be an explanation for the non-significance of preventable factors between Iranians and Afghans. Another limitation is improper documentation. For instance, no records from the first institute or from professionals who referred patients to the tertiary hospitals were found, and antenatal cards were mostly unavailable. The quality of pre-hospital care was assessed based on the information extracted from the hospital admission notes and the additional input that was given by a few survivors and could thus be underestimated. We were unable to make contact with a majority of near-miss mothers for interviews. Furthermore, an interviewer who was not an obstetric professional could have received other responses or provided other input to the audits. Two auditors worked as consultant obstetricians at the study sites, one in tertiary and one in the secondary hospital, and this may have impacted on their judgements of care quality at their hospitals. However, MNM cases were presented anonymously at panels and auditors were unaware of the cases’ nationality or the place in which they were treated.

## Conclusion

Inequalities in maternal care between Iranians and Afghan migrants were identified. The majority of near-miss events were considered preventable and audits suggested areas for improvements. The results may draw the attention of policy makers for adopting a maternal morbidity surveillance and response system to tackle care disparities and address obstacles in obstetrics. Afghan women affected by many socioeconomic and humanitarian challenges that should gain additional attention within antenatal visits.

## Abbreviations

AOR: Adjusted odds ratio
BMI: Body mass index
CI: Confidence interval
CS: Caesarean section
CTG: Cardiotocography
MNM: Maternal near miss
OR: Odds ratio
WHO: World health organization

## References

[1] Wahlberg Å, Rööst M, Haglund B, Högberg U, Essén B. Increased risk of severe maternal morbidity (near-miss) among immigrant women in Sweden: a population register-based study. BJOG. 2013;120(13):1605–11. 10.1111/1471-0528.12326 23786308

[2] Zwart JJ, Jonkers MD, Richters A, Ory F, Bloemenkamp KW, Duvekot JJ (2011). Ethnic disparity in severe acute maternal morbidity: a nationwide cohort study in the Netherlands. Eur J Public Health.

[3] Almeida LM, Caldas J, Ayres-de-Campos D, Salcedo-Barrientos D, Dias S (2013). Maternal healthcare in migrants: a systematic review. Matern Child Health J.

[4] Lindquist A, Noor N, Sullivan E, Knight M (2015). The impact of socioeconomic position on severe maternal morbidity outcomes among women in Australia: a national case-control study. BJOG.

[5] Esscher A, Binder-Finnema P, Bodker B, Högberg U, Mulic-Lutvica A, Essén B (2014). Suboptimal care and maternal mortality among foreign-born women in Sweden: maternal death audit with application of the ‘migration three delays’ model. BMC Pregnancy Childbirth.

[6] The World Bank. Iran overview. http://www.worldbank.org/en/country/iran/overview. Accessed 4 Dec 2016.

[7] United Nations High Commissioner for Refugees. Iran Factsheet. 2016. http://www.unhcr.org/protection/operations/50002081d/iran-fact-sheet.html. Accessed 7 Nov 2016.

[8] UNICEF. Iran, Islamic Republic of. Statistics 2012. http://unicef.org/infobycountry/iran_statistics.html. Accessed 4 Dec 2016.

[9] World Health Organization. Trends in maternal mortality: 1990 to 2015. http://www.who.int/reproductivehealth/publications/monitoring/maternal-mortality-2015/en/. Accessed 6 Nov 2016.

[10] Country Cooperation Strategy for WHO and the Islamic Republic of Iran. 2010–2014. http://www.who.int/countries/irn/en/. Accessed 6 Nov 2016.

[11] Mohammadi S, Essén B, Fallahian M, Taheripanah R, Saleh Gargari S, Källestål C (2016). Maternal near-miss at university hospital with cesarean overuse: an case-control study. Acta Obstet Gynecol Scand.

[12] van Roosmalen J, Zwart J (2009). Severe acute maternal morbidity in high-income countries. Best Pract Res Clin Obstet Gynaecol.

[13] World Health Organization. Evaluating the quality of care for severe pregnancycomplications: The WHO near miss approach for maternal health. 2011. http://apps.who.int/iris/bitstream/10665/44692/1/9789241502221_eng.pdf. Accessed 8 Feb 2017.

[14] Bahadori FH, Hakimi M, Heidarzadeh M (2013). The trend of caesarean delivery in the Islamic Republic of Iran. East Mediterr Health J.

[15] Mantel GD, Buchmann E, Rees H, Pattinson RC (1998). Severe acute maternal morbidity: a pilot study of a definition for a near-miss. Br J Obstet Gynaecol.

[16] Merali HS, Lipsitz S, Hevelone N, Gawande AA, Lashoher A, Agrawal P (2014). Audit-identified avoidable factors in maternal and perinatal deaths in low resource settings: a systematic review. BMC Pregnancy Childbirth.

[17] World Health Organization. Quality of care: a process for making strategic choices in health systems. WHO Library Cataloguing-in-Publication Data. 2006. http://www.who.int/iris/handle/10665/43470. Accessed 8 Feb 2017.

[18] Binder P, Borné Y, Johnsdotter S, Essén B (2012). Shared language is essential: communication in a multiethnic obstetric care setting. J Health Commun.

[19] Karlsen S, Say L, Souza JP, Hogue CJ, Calles DL, Gülmezoglu AM (2011). The relationship between maternal education and mortality among women giving birth in health care institutions: analysis of the cross sectional WHO Global Survey on Maternal and Perinatal Health. BMC Public Health.

[20] Rööst M, Jonsson C, Liljestrand J, Essén B (2009). Social differentiation and embodied dispositions: a qualitative study of maternal care-seeking behaviour for near-miss morbidity in Bolivia. Reprod Health.

[21] van Dillen J, Mesman JA, Zwart JJ, Bloemenkamp KW, van Roosmalen J (2010). Introducing maternal morbidity audit in the Netherlands. BJOG.

[22] Olmedo B, Miranda E, Cordon O, Pettker CM, Funai EF (2014). Improving maternal health and safety through adherence to postpartum hemorrhage protocol in Latin America. Int J Gynaecol Obstet.

[23] Pattinson RC, Say L, Makin JD, Bastos MH (2005). Critical incident audit and feedback to improve perinatal and maternal mortality and morbidity. Cochrane Database Syst Rev.

[24] Mohammadi S, Källestål C, Essén B (2012). Clinical audits: a practical strategy for reducing cesarean section rates in a general hospital in Tehran. Iran J Reprod Med.

[25] The FIGO committee and working group publications. FIGO committee for the Ethical Aspects of Human Reproduction and Women’s Health. Ethical issues in obstetrics and gynaecology. 2012. http://figo.org/figo-committee-and-working-group-publications. Accessed 8 Feb 2016.

[26] Wittevenn T, de Koning I, Bezstarosti H, van den Akker T, van Roosmalen J (2016). Bloemen kamp KW. Validating the WHO maternal near miss tool in a high-income country. Acta Obstet Gynecol Scand.

